# Longitudinal studies of bipolar patients and their families: translating findings to advance individualized risk prediction, treatment and research

**DOI:** 10.1186/s40345-024-00333-y

**Published:** 2024-04-12

**Authors:** Anne Duffy, Paul Grof

**Affiliations:** 1 Department of Psychiatry, Queen’s University, Kingston, ON Canada; 2 Department of Psychiatry, University of Oxford, Oxford, UK; 3 Department of Psychiatry, University of Toronto, Toronto, ON Canada

**Keywords:** Bipolar disorder, Lithium response, Lithium non-response, Family studies, Genetic studies, Biomarkers, High-risk offspring, Pharmacotherapy, Longitudinal studies, Individualized treatment

## Abstract

**Background:**

Bipolar disorder is a broad diagnostic construct associated with significant phenotypic and genetic heterogeneity challenging progress in clinical practice and discovery research. Prospective studies of well-characterized patients and their family members have identified lithium responsive (LiR) and lithium non-responsive (LiNR) subtypes that hold promise for advancement.

**Method:**

In this narrative review, relevant observations from published longitudinal studies of well-characterized bipolar patients and their families spanning six decades are highlighted. DSM diagnoses based on SADS-L interviews were decided in blind consensus reviews by expert clinicians. Genetic, neurobiological, and psychosocial factors were investigated in subsets of well-characterized probands and adult relatives. Systematic maintenance trials of lithium, antipsychotics, and lamotrigine were carried out. Clinical profiles that included detailed histories of the clinical course, symptom sets and disorders segregating in families were documented. Offspring of LiR and LiNR families were repeatedly assessed up to 20 years using KSADS-PL format interviews and DSM diagnoses and sub-threshold symptoms were decided by expert clinicians in blind consensus reviews using all available clinical and research data.

**Results:**

A characteristic clinical profile differentiated bipolar patients who responded to lithium stabilization from those who did not. The LiR subtype was characterized by a recurrent fully remitting course predominated by depressive episodes and a positive family history of episodic remitting mood disorders, and not schizophrenia. Response to lithium clustered in families and the characteristic clinical profile predicted lithium response, with the episodic remitting course being a strong correlate. There is accumulating evidence that genetic and neurobiological markers differ between LiR and LiNR subtypes. Further, offspring of bipolar parents subdivided by lithium response differed in developmental history, clinical antecedents and early course of mood disorders. Moreover, the nature of the emergent course bred true from parent to offspring, independent of the nature of emergent psychopathology.

**Conclusions:**

Bipolar disorders are heterogeneous and response to long-term lithium is associated with a familial subtype with characteristic course, treatment response, family history and likely pathogenesis. Incorporating distinctive clinical profiles that index valid bipolar subtypes into routine practice and research will improve patient outcomes and advance the development and translation of novel treatment targets to improve prevention and early intervention.

## Background

The observation of alternating depressive and manic episodes recurring over time within the same individual thus representing a singular illness process dates to antiquity(Mason et al. 2016). European scholars continued to describe various forms of this recurrent mood disturbance (i.e. folie circulaire, cyclothymia) through the 16th and 17th centuries (Sedler 1983; Angst and Sellaro 2000). In 1895, Kraepelin coined the term *manic depressive insanity* describing an episodic illness characterized by a recurrent remitting course (with a free interval or spontaneous good quality remission) and better long-term prognosis compared to dementia praecox (i.e. schizophrenia)(Kraepelin 1899, 1902). More recent diagnostic criteria (i.e. post DSM-III), have emphasized the importance of the manic episode and shifted attention from the hallmark recurrent remitting course central to the Kraepelinian manic depressive insanity construct (Grof et al. 1995). Over time, the bipolar diagnostic construct has broadened and heterogeneity increased, related not only to changes in criteria, but also in diagnostic practice. That is, diagnosis shifted to a cross-sectional assessment of presenting psychopathology in more heterogeneous patient populations focused on a list of non-specific criterion symptoms (i.e. reduced sleep, increased energy/agitation, thought disorder). This approach failed to incorporate context, developmental timing, and the pervasive nature of symptoms across settings, as part of a comprehensive clinical assessment that relied upon longitudinal observation and collateral history (Duffy et al. 2018; Grof et al. 1995). As Kendler so aptly put it “*since DSM-III, our field has moved toward a reification of DSM that implicitly assumes that psychiatric disorders are actually just the DSM criteria. That is, we have taken an index of something for the thing itself* “(Kendler 2016).

As heterogeneity of patients diagnosed with bipolar disorder increased, effectiveness of lithium as stabilizing agent seemingly diminished, and the search for genetic and neurobiological correlates and new treatment targets stalled (Manchia et al. 2013b; Hodgson et al. 2017; Craddock and Owen 2010). Further, despite intensive research over several decades into a variety of stabilizing treatments including second generation antipsychotics and anticonvulsants, the outcome for bipolar patients has not substantively improved (Geddes and Miklowitz 2013), and arguably may have worsened (Post et al. 2016). Published treatment guidelines continue to recommend lithium as the gold standard maintenance treatment given the robust independently replicated efficacy data (Geddes and Miklowitz 2013; Volkmann et al. 2020; Fountoulakis et al. 2022). However, evidence demonstrates that lithium continues to be underutilized (Singh et al. 2024; Malhi et al. 2023), in part related to concerns about the need for serum lithium monitoring to minimize risk of long-term adverse effects, set against the appeal (despite relatively weaker evidence) of newer pharmacological options (Fountoulakis et al. 2022; Volkmann et al. 2020; Singh et al. 2024; Kessing 2024). Notwithstanding diagnostic challenges, there is recognition of the pressing need to identify more homogeneous subtypes of bipolar disorder that would lend themselves to the selection of a specific stabilizing treatment for individual patients to improve patient outcomes and map to underlying pharmacological targets and neural mechanisms (Geddes and Miklowitz 2013; Malhi and Geddes 2014).

In line with Kraepelin’s approach to differentiating major psychiatric illnesses based on longitudinal observation of the natural history, substantive findings from prospective studies of well-characterized bipolar patients and their family members have identified replicable subtypes (Grof et al. 2009; Alda 2017; Grof 2003). This selective narrative review summarizes published evidence and some unpublished observations from longitudinal studies over decades of bipolar adults subtyped by their clinical profile, in conjunction with systematic studies of their adult family members and offspring. The aim is to provide a succinct overview of the evidence from these longitudinal studies that could be translated to advance risk prediction, early accurate diagnosis, and individualized pharmacotherapy. These findings could also inform our understanding of the pathogenesis of bipolar subtypes and ultimately advance the identification and development of novel treatments mapped to developmental stage of illness (Duffy and Carlson 2013; Duffy 2015; Grof et al. 2009).

### Key findings and observations from the adult studies

Observing both the untreated and treated illness course, investigating multigenerational families, and differentiating bipolar subtypes enriches the understanding of the onset of bipolar disorders and prediction of effective long-term treatment. Systematic long-term studies confirm the importance of bipolar subtypes identified on the basis of each patient’s clinical profile – information available in routine clinical practice (Alda 2004; Grof et al. 2009). To determine an effective treatment, it is essential to base the decisions on verified (as much as possible) detailed information about the patient’s developmental history, clinical course, symptoms and their context, and family history – and not just rely upon DSM diagnostic criteria (Duffy et al. 2018).

#### Stabilizer (tong-term) response

We started to investigate adults with recurrent mood disorders in 1962 and expanded this research in the setting of a clinical program (inpatient and outpatient) in 1968. Lithium maintenance had started to be prescribed to patients in December 1966 (Angst et al. 1969). At that time, many patients who came to us for help had been severely ill for decades. Each year they were spending many months in hospital with acute illness recurrences. Achieving full stabilization with lithium seemed for such patients and their families rather miraculous (Grof 1999, 2010).

Such a dramatic change generated pressing questions as to the mechanism underlying this full recovery and how to best identify patients who would achieve full remission on lithium. In an attempt to answer these questions, we performed clinical, family, genetic, and neurobiological studies for the next four decades (Grof 2010). The main strategy used was comparing two groups of bipolar patients; those who were excellent responders to lithium stabilization to those who were clear non-responders. In the adult bipolar cohort, the patients were followed up regularly for various lengths of time, spanning 3 to 55 years (Grof 2010). The relatively shorter follow-ups were mostly for patients who did not respond to lithium or in those with a low recurrence risk (i.e. long intervals between episodes).

Only carefully diagnosed bipolar patients who could be clearly identified as either lithium responders (LiR) or as lithium non-responders (LiNR) could provide the answers (Grof et al. 1993). In bipolar patients with a lower recurrence risk, natural remission is easily confused with treatment response. Recurrence risk is capricious and also varies according to the bipolar subtype (Angst 1978). It is easy to confuse poor compliance with treatment non-response. Fortunately, jointly with Jules Angst we studied lifelong histories of untreated patients during the previous decade and were aware of these potential confounds (Angst et al. 1973). By 1978, it became clear from systematic prospective observation of treatment response that there was at least three major bipolar subtypes differentiated by clinical characteristics and family histories (Angst 1978; Alda 2004). In these studies, bipolar patients had to have a high recurrence risk prior to starting lithium treatment and were identified as responsive to lithium only if they exhibited no illness recurrences for a sufficient observation period (i.e. exceeding 3 years or 2 cycle lengths) with evidence of acceptable lithium serum levels. On the other hand, lithium non-response was determined by two acute recurrences (either manic or major depressive episodes) over the observation period, despite evidence of acceptable serum lithium levels (Grof et al. 1993).

Compared to controls, bipolar patients showed marked abnormalities in virtually all neurobiological investigations performed (Grof 2010). However, differences in neurobiological markers between LiR and LiNR bipolar patients were, despite the striking differences in clinical profiles, either minor or too expensive to identify and thus not helpful for predicting lithium response in clinical practice. Eventually, when all data were included in multivariate analyses, only the individual clinical profiles sufficiently and significantly separated the two subgroups (Alda 2017; Grof 2006b). Lithium responders had recurrent course, with free intervals (good quality of remissions) showing full absence of psychopathology during clinical assessment and on psychological testing. Comorbid conditions were relatively infrequent. Similarly, in their families there was only episodic psychopathology present (Grof 2006a). If a clinician had solid information about the patient’s clinical profile including developmental history, onset and clinical course, and family history, in over 80% of cases it was possible to predict the response or non-response to lithium treatment (Grof et al. 1983). For more accurate prediction of the specific stabilizing effect, it is necessary to differentiate between a true lithium response (full remission and cessation of recurrences) and antipsychotic-like partial response (with evidence of some improvement but chronic fluctuating clinically significant symptoms)(Grof 1998). We also observed that non-responders to lithium benefited from other mood stabilizers and had different clinical profiles compared to LiR (Grof 2003, 2004; Passmore et al. 2003).

#### Bipolar subtypes

The natural (untreated) bipolar course and recurrence risk vary significantly between individuals and differ between bipolar subtypes (Angst 1978; Grof 2003). This variability makes it challenging to accurately evaluate the recurrence risk and the true outcome of stabilizing treatment for individual patients. Wider interest in the untreated bipolar course started emerging coincident with stabilizing treatment making it into routine clinical practice, further complicating the assessment of recurrence risk (International Consortium on et al. 2018; Zis 1979).

Bipolar subtypes can be identified from the patient’s full clinical profile, and not just from the assessment of symptom clusters. Each bipolar subtype has a distinctive clinical course, family history, treatment response, and prognosis. Therefore, it is likely and accumulating evidence supports that different bipolar subtypes (indexed by the clinical profile) have important differences in pathogenesis (Alda 2004, 2017).

The strongest differentiating feature of the lithium responsive subtype is a fully remitting clinical course (Nunes et al. 2020; Grillault Laroche et al. 2020). Unfortunately, clinicians are generally well trained in identifying acute psychopathology, but not assessing normative mental health or quality of full remission. It is easy to mistake a degree of functional recovery for a complete remission and return to premorbid functioning. Patients with some functional recovery usually report no or little distress, and after an acute episode return to their work and family. However, on closer assessment they have identifiable fluctuations in sleep, mood, anxiety, and cognition not typically identified, but indicative of residual active, non-fully remitted illness. Such patients are often unnecessarily maintained on lithium without the benefit of full stabilization*, exposed to the risk of adverse effects, and lose the opportunity to possibly benefit more from one of the other mood stabilizing alternatives (Grof 2003; Grof et al. 1993). *there can be non-specific benefits from lithium including an anti-suicidal effect, even for patients who do fully remit (i.e. are not excellent lithium responders in terms of stabilization).

#### Clustering of in families

The other important observation has been the clustering of psychiatric disorders in the families of bipolar patients. We observed this clustering working clinically with patients over the long-term in the 1970s. When molecular genetic technology made it possible to systematically study associated genes in the laboratory, we initiated a genetic study of families led by Martin Alda as the principal investigator (Alda 1997). Eventually, this work contributed to an international collaboration with the IGSLi group (https://www.igsli.org/) (Alda et al. 2000; Turecki et al. 2001; Turecki et al. 1999, 1998; Turecki et al. 2000). In more recent consortia collaborations the effort of collecting genetic data in well-characterized families over three decades has been successful in demonstrating the importance of the familial clustering to advance understanding of treatment response and genetic underpinnings (Nunes et al. 2020, 2021).

The observation that treatment response runs in the families of patients with bipolar disorders has occasionally been reported (Pare and Mack 1971; Grof et al. 2002), but is difficult to study systematically. Traditionally, affected members of the same family are usually treated by different physicians and treatment guidelines do not consider the informative value of familial response in treatment selection. Over three decades ago, we recruited a large cohort of families identified by a well-characterized bipolar patient and then assessed, followed, and treated family members who became affected (Alda 1997). The families varied in size; some exceeded one hundred investigated relatives of the proband. The observations based on these families demonstrated how psychiatric disorders cluster in such families, with LiRs having relatives with episodic disorder and LiNRs having relatives with non-episodic disorders (Duffy and Grof 2001). The responsiveness to a particular type of mood stabilizer was also observed to breed true or cluster in families (Grof et al. 2002).

An unexpected, interesting pattern emerged in which the affected relatives met diagnostic criteria for various psychiatric disorders, but showed striking uniformity in the clinical profile, including the nature of the clinical course and responses to acute and stabilizing pharmacotherapy. This observation suggests that the response to pharmacotherapy appears relatively independent of the specific psychiatric diagnoses in affected relatives. While the spectrum of disorders segregating in the families differed between LiR and antipsychotic-responsive (LiNR) families, the clinical profile within families remained the same and differentiated these subtypes (Grof 2010; Duffy and Grof 2001). These observations raise an uncomfortable possibility that patients with a comparable underlying neurobiological disturbance may present with various symptom sets and meet the criteria for different or event multiple diagnoses based on current taxonomy. Furthermore, these observations raise the question of whether a fully comprehensively evaluated family history should be included in the future diagnostic criteria for mood disorders and incorporated into treatment guidelines.

**Fig. 1:**
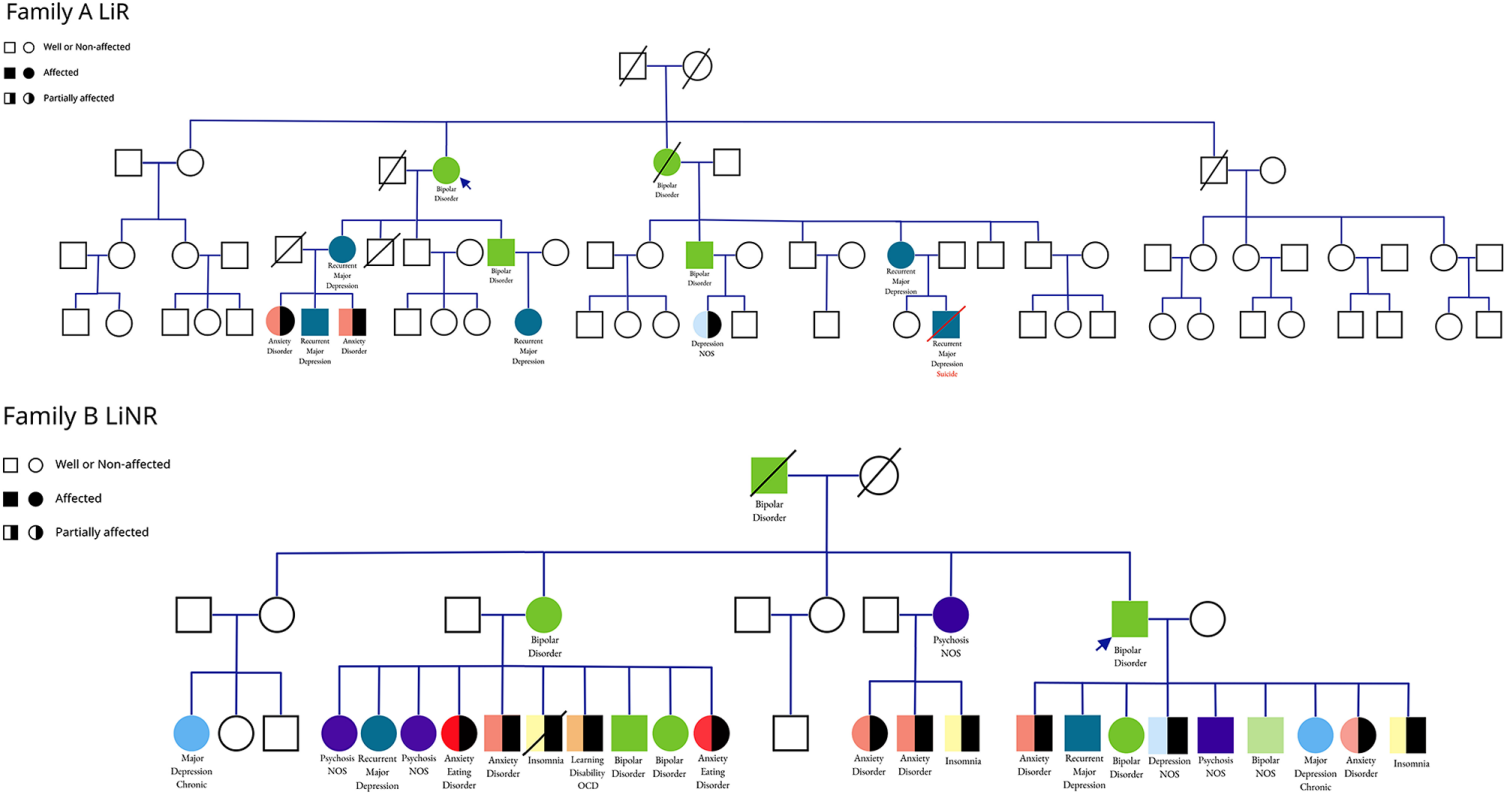
Phenotypic Spectrum in Families of a Lithium Responsive (LiR) and Lithium Non-Responsive (LiNR) Bipolar Proband. **(A)** Lithium Responsive Family. **(B)** Lithium Non-Responsive Family (antipsychotic responder)

To illustrate the findings summarized above, we present two large pedigrees; one identified through a lithium responsive bipolar proband and the other from a lithium non-responsive (antipsychotic responsive) bipolar proband (see Fig. 1a and b).

### Key findings from offspring studies

Longitudinal studies of the children of prospectively well-characterized and systematically treated bipolar parents over the peak period of risk (childhood through adolescence and into adulthood) have provided important insights into the developmental history and early course of emerging bipolar disorder (Duffy et al. 2017b; Duffy 2015). While there is some overlap in non-specific clinical antecedents across high-risk offspring subgroups, there were consistent and striking differences in clinical trajectories observed between the offspring of LiR and LiNR parents (Duffy et al. 2019). Specifically, offspring of a bipolar parent with an excellent response to long-term lithium (LiR) were observed to have normal or gifted (academically, socially) early childhood development comparable to the children of well parents (Duffy et al. 2007a, 2010). By contrast, offspring of a parent with a LiNR bipolar disorder manifest a number of academic, cognitive and social problems similar to the observations of children of parents with schizophrenia (Duffy 2012). This observation aligns with findings from the Danish VIA study which reported widespread neurocognitive deficits in children at familial risk of schizophrenia and an absence of neurocognitive deficits in children at familial risk of bipolar disorder (narrowly defined based on ICD criteria) comparable to that of controls (Hemager et al. 2018).

Clinical presentations at both the syndrome and clinically assessed symptom levels suggest that children of both LiR and LiNR bipolar parents manifest childhood anxiety and sleep disorders earlier and at a higher rate than offspring of well parents (controls) (Duffy et al. 2019). Further, in both high-risk offspring subgroups these early childhood non-mood presentations predicted a higher risk of major mood episodes (depressive and manic/hypomanic) in later adolescence (Duffy et al. 2010, 2013). Interestingly, the clinical course of these early non-mood antecedents differed between offspring subgroups; with episodic and remitting anxiety and/or sleep disorders in children of LiRs, and chronic or only partially remitting non-mood antecedent presentations in children of LiNRs (Duffy et al. 2002). This observation supports that in children at confirmed familial risk for bipolar disorder, “non-specific” childhood antecedents have the same clinical course as the subtype of parental bipolar disorder (LiR vs. LiNR). Implications of this observation include that these childhood antecedents may index the predisposition for the underlying bipolar subtype in the context of an immature developing brain, rather than being a non-specific indicator of a shared or pluripotential vulnerability as others have postulated (Duffy et al. 2017a; McGorry et al. 2018; Duffy and Carlson 2013).

Prospective offspring studies have clearly demonstrated the preponderance of depressive episodes in the early course of evolving bipolar disorder across high-risk subtypes (Mesman et al. 2013; Duffy et al. 2017b). Infact, bipolar disorder debuted as a depressive episode (rather than manic or hypomanic episode) in over 85% of prospectively observed offspring at familial high-risk (Duffy et al. 2017b, 2019; Mesman et al. 2013). Observations of symptom-level mood psychopathology in high-risk offspring support that internalizing (anxiety, low mood, sensitivity, mood lability) and activated symptoms (high energy, euphoria) antecede major mood episodes and predict the onset of recurrent major mood disorder (Duffy et al. 2019; Hafeman et al. 2017; Keown-Stoneman et al. 2021). In summary, the striking differences in the emergent course of evolving bipolar related mood disorders between the offspring of parents with either a LiR and LiNR subtype include that: (i) offspring of LiRs show an episodic spontaneously remitting course with good quality of spontaneous remission and stable high functioning over development; (ii) offspring of LiNRs in contrast show a layering of psychopathology that does not fully remit and reduced global functioning over time (residual symptoms observed include paranoid thinking, social withdrawal, anxiety and mood symptoms, cognitive dysfunction); (iii) depressive episodes were observed to dominate the early course of bipolar disorder in both LiR and LiNR offspring subgroups, but more so in the offspring of LiRs, and (iv) while most of the first five major mood episodes in both offspring subgroups did not feature psychotic symptoms (less than 30%), the offspring of LiRs were significantly less likely to manifest psychotic symptoms in mood episodes compared to the offspring of LiNRs, despite severity of acute mood episodes being comparable between the groups (Duffy et al. 2014, 2019).

When comparing age of onset, nature of the clinical course, quality of remission, functioning over time and response to mood stabilizers in open prospective studies, offspring shared the clinical profile of their affected bipolar parent (Duffy et al. 2002, 2019). That is offspring of LiRs themselves had an episodic remitting course of mood disorder that tended to respond to lithium maintenance (Duffy et al. 2007b). Furthermore, we observed that when non-mood childhood antecedents such as anxiety and depression required pharmacotherapy and did not respond to guideline driven treatment (i.e. SSRI), the stabilizer that benefitted the parent also benefitted the offspring (Duffy and Grof 2018). Finally, when offspring did meet criteria for a bipolar spectrum disorder and required pharmacotherapy, the stabilizer that benefitted the parent also tended to benefit the affected offspring (Duffy et al. 2007b, 2009b). Most offspring (and their parents) did not wish to continue pharmacotherapy long-term, but in the short to medium-term the pharmacological intervention selected on the basis of familial response was observed to be effective and generally well tolerated at minimal effective doses (Duffy et al. 2007b).

## Discussion

A substantive body of clinical and basic research on bipolar disorders has accumulated in the literature over several decades. Numerous clinical trials of pharmacotherapies have been completed and several decades of treatment guidelines have been published. Nonetheless, the outcome for bipolar patients has not substantively improved and the pathogenesis remains insufficiently understood. The focus on symptom-level psychopathology championed in RDoC (Cuthbert and Insel 2013) was meant to cut through the stalemate related to the heterogeneity inherent in psychiatric diagnostic categories and better map psychopathology to underlying neurobiology. However, this approach has not yielded the hoped for progress, in part because symptoms are open to interpretation, non-specific, and require the predictive lens of developmental and family history to sharpen the focus and confer clinical meaning (Duffy et al. 2018). The field is ripe for change and one promising evidence-based path forward is a focus on homogeneous and familial illness subtypes subsumed in current broad heterogeneous diagnostic categories (Hodgson et al. 2017).

In this selective review of longitudinal studies of systematically prospectively assessed and treated bipolar patients and their families spanning six decades, we have attempted to summarize the relevant evidence supporting valid bipolar subtypes identifiable on the basis of a distinctive clinical profile. The identified bipolar subtypes have important implications for improving risk prediction, selection of stabilizing treatment, as well as significant potential for advancing applied and basic research. The clinical profile that identifies these bipolar subtypes should include what in traditional medicine led to charting exemplars of recognizable natural history and illness trajectories, which in turn informed clinical staging models and biomarker research, leading to advances in understanding pathogenic mechanisms, paving the way to developing gold standard diagnostic tests and progressing the identification of novel treatment targets. Solid evidence from earlier longitudinal clinical studies have demonstrated that properly selected bipolar patients can be stabilized on lithium monotherapy for decades (Berghofer et al. 2013). Moreover, lithium has very different neurobiological targets and activities compared to other stabilizing agents, which suggests the lithium responders may have a distinctive pathogenesis.

The clinical profile requires a detailed developmental history, description of the onset, clinical course and symptom sets , along with a carefully assessed family history of psychiatric illness and where possible familial response to treatment. By using the clinical profiles of a classical manic-depressive illness responsive to lithium stabilization and the other lithium non-responsive (anticonvulsant and antipsychotic) profiles, the accuracy of individualized prediction of prognosis and treatment response will improve and research into the pathogenesis of bipolar disorder will advance. A comprehensive clinical assessment of the patient, going beyond the symptoms and refraining from defaulting to a checklist approach to confirm a psychiatric diagnosis is not new and goes to the heart of the principles of evidence-based medicine (Duffy et al. 2018; Kendler 2016; ) - that is using all of the available informative evidence in the formulation of a diagnosis and treatment plan. A developmental approach to map the evolution of psychopathology and a detailed family history based on direct interview of available members and/or multiple informants incorporated into the diagnostic formulation and risk prediction is especially important in young people, who often manifest non-specific psychopathology as part of the early natural history of emergent disorder (Duffy and Carlson 2013; Duffy 2015).

Time spent in completing a detailed assessment of a patient using all available clinical information and multiple informants which may extend over a period of time, is a solid investment that in our experience pays dividends in terms of the quality of remission and reduction of adverse events and recurrences achieved in treatment, along with providing a better understanding of the illness under investigation. While this type of detailed diagnostic assessment is not well-suited to emergency care, it is appropriate and feasible in specialty mood disorders inpatient and outpatient settings as our work has shown. By working with patients over time, we have learned to discern improvement from remission, identify familial patterns of illness segregation and treatment response, and hav been better able to differentiate contextual psychosocial perturbations from illness symptoms. As a result of this longitudinal systematic clinical observation, we have confirmed and built on Angst’s earlier work (Angst 1978), characterizing three distinctive subtypes of bipolar disorder with real potential to advance practice and research, if appropriately adopted and incorporated into treatment guidelines and research designs.

Limitations and caveats include that clinical profiles may be difficult to discern in patients for whom accurate family history and clinical course information is not obtainable or the natural illness course is altered or masked through treatment with multiple medications over a long period of time. Further, even in excellent lithium responders, breakthrough symptoms (i.e. difficulty falling asleep, minor mood symptoms) are not uncommon in active periods of illness (which if untreated would manifest as acute recurrences) during the early days of starting lithium treatment and may require short-term additional symptomatic treatment (i.e. sleeping medication). Spontaneous remission and treatment non-compliance can be misinterpreted as response or non-response to a trial of lithium, respectively. Further, a certain proportion of patients will require combination stabilizing treatment given medical comorbidities or to gain the non-specific benefits of lithium. That is, lithium has a variety of benefits that go beyond the specific stabilizing effect and use should therefore not be entirely curtailed to stabilization in patients with a lithium responsive bipolar profile (Grof and Grof 1990; Grof and Muller-Oerlinghausen 2009). Finally, the prediction of lithium response based on clinical profiles outlined here has not as yet been tested in a large randomized controlled trial. Nonetheless, the outcome defining response or non-response to lithium was based on long-term prospective observation requiring full remission or full episode recurrence with proof of adequate serum lithium levels, respectively. Furthermore, family history and clinical course were collected as part of systematic research studies using all available clinical and research materials (including FH-RDC interviews of relatives) and based on blind consensus reviews by expert clinicians.

The implications of the findings outlined here include that (i) there are distinctive clinical profiles indexing valid bipolar subtypes that predict for a specific response or non-response to lithium stabilization; (ii) non-responders to lithium may benefit from other stabilizers based on their clinical profiles (anticonvulsant and antipsychotic); (iii) clinical profiles indexing bipolar subtypes should be incorporated in routine practice and research; and (iv) diagnosis based on the full clinical profile including a detailed developmental and family history, clinical course, and symptom sets, and ruling out of other medical and psychiatric problems should replace the current approach that relies on cross-sectional assessment of symptoms.

## Methods

This manuscript is a selective review of longitudinal published clinical and genetic studies and observations that have contributed to the validation of bipolar subtypes identified on the basis of distinctive clinical profiles. Shared aspects of the previously published methods from studies included in this narrative review are briefly outlined below.

### Adult bipolar patients

Bipolar patients were initially assessed in a comprehensive consultation and based on diagnosis and clinical need admitted for care and long-term follow-up in university affiliated hospital-based inpatient and outpatient mood disorders clinical programs.

Since 1978, DSM diagnoses were based on SADS-L interviews and decided by consensus reviews of all available clinical material by expert clinicians. The patients were followed up prospectively, regularly as required, on average every few months. The length of observation varied from three to fifty-five years. As new treatment and neuroscience approaches emerged, research questions were addressed in separately designed and funded studies, recruiting suitable consenting patients from these clinical programs. Each study was reviewed for ethical approval by the local hospital research ethics board.

The main strategy for clinical and genetic studies that emerged was based on identifying from among the bipolar patients, excellent responders to long-term trials of lithium as a monotherapy stabilizer and comparing outcomes to clear non-responders to lithium stabilization. As outlined in the published literature, all patients had a bipolar diagnosis and a high recurrence risk prior to lithium treatment. Whilst on lithium treatment for a minimum of 3 years, responders had no recurrences of any polarity and non-responders had 2 recurrences with documented adequate lithium levels (Grof et al. 1993, 2009). Neurobiological correlates and genetic factors were investigated in subsets of lithium-responsive and lithium-nonresponsive bipolar patients (Turecki et al. 1998; Alda et al. 2000). In later family and genetic studies, all available consenting family members were interviewed using SADS-L interviews and diagnosis was based on blind consensus reviews by expert clinicians. For family members not assessed directly family history from multiple informants was collected using FH-RDC interview format and diagnosis based on blind consensus reviews. For affected family members not treated in our clinical research program, a probabilistic determination of likelihood of lithium response was made on the basis of clinical profiles taking into account completeness of remission, duration of treatment, recurrence risk, and use of any other stabilizers (Manchia et al. 2013a; Nunes et al. 2020).

### Offspring

Offspring of bipolar parents were recruited into this dynamic, longitudinal, naturalistic cohort study from families systematically longitudinally assessed and participating in the clinical and genetic research studies described above and in prior publications (Duffy et al. 1998, 2002, 2014, 2019). Briefly, assenting/consenting offspring ages 5–25 years from bipolar parents subtyped on the basis of parental excellent response or non-response to lithium prophylaxis (LiR vs. LiNR) were assessed blindly to family and study group using semi-structured research interviews following KSAD-PL format conducted by an expert child and adolescent psychiatrist.. Research interviews and all available clinical information (psychoeducational testing, prior clinical consult notes, hospitalizations, developmental history completed by parents) was reviewed by a panel of expert clinicians and DSM diagnosis based on blind consensus review. Later a control group of offspring assessed blind to study groups using KSADS-PL format interviews of well-parents themselves assessed by SADS-L interviews was included in the study. All offspring from LiR, LiNR, and Control groups were followed on average annually using KSADS-PL interviews and blind consensus reviews for up to two decades. Clinically significant symptoms were also assessed against published operational criteria (Duffy et al. 2019) and validated age appropriate parent andself-report symptom measures were completed along with measures of theoretically important psychosocial risk factors at each research visit (Duffy et al. 2001, 2007c, 2009a, 2012, 2013, 2019; Doucette et al. 2013; Goodday et al. 2017, 2018a, b, 2019). At last reported observation, the high-risk offspring (*n* = 279) mean duration of follow-up was 7.7 years (SD 5.28), ranging from 1 to 21 years. Attrition over this period was less than 5% (Duffy et al. 2019).

## Data Availability

No datasets were generated or analysed during the current study.

## Abbreviations

DSM: Diagnostic and Statistical Manual
FH-RDC: Family History – Research Diagnostic Criteria
LiR: lithium responsive bipolar subtype
LiNR: lithium non-responsive bipolar subtype
KSADS-PL: Kiddie Schedule for Affective Disorders – Lifetime
SADS-L: Schedule of Affective Disorders - Lifetime
